# Increasing Need for Uniqueness in Contemporary China: Empirical Evidence

**DOI:** 10.3389/fpsyg.2018.00554

**Published:** 2018-05-08

**Authors:** Huajian Cai, Xi Zou, Yi Feng, Yunzhi Liu, Yiming Jing

**Affiliations:** ^1^Key Laboratory of Behavioral Sciences, Institute of Psychology, Chinese Academy of Sciences, Beijing, China; ^2^London Business School, London, United Kingdom

**Keywords:** need for uniqueness, China, cultural change, individualism-collectivism, social change

## Abstract

Past research has documented various cultural and psychological changes in contemporary China. In two studies, we examine how Chinese people’s need for uniqueness (NFU) also has changed. In Study 1, we found a significant cross-generational increase in Chinese participants’ self-reported NFU. In Study 2, we sampled the names of Chinese newborn babies over the last five decades and found that parents have been increasingly likely to use unique characters to name their children. These findings suggest that the NFU has been rising in China, a historically collectivistic-oriented society. Theoretical and practical implications of our findings were discussed.

## Introduction

Unprecedented economic growth and social transformation have led to substantial changes in Chinese people’s psychology. For instance, past research has found that a variety of individualistic values (e.g., autonomy; Xu and Hamamura, 2014) and traits (e.g., narcissism; Cai et al., 2011) have been rising in China, a historically collectivistic-oriented society. In this article, we focused on a possible change in Chinese people’s need for uniqueness (NFU), a psychological need that is typically high in the West but assumed to be relatively low in the East (Markus and Kitayama, 1991). Specifically, we investigated whether the NFU has been increasing in China. Examining this issue will enrich our understanding of Chinese people’s psychological change.

### Need for Uniqueness

The NFU refers to “a positive striving for differentness relative to other people” (Snyder and Fromkin, 1977, p. 518). People with a high NFU value non-conformity (Asch, 1956) and desire to be unique, separable, and distinct from “the masses” (Snyder and Fromkin, 1980). People may express their NFU in diverse ways such as through creativity (Fromkin, 1968; unpublished), subjective experiences (Fromkin, 1970), attitudes (Weir, 1971; unpublished), judgments (Duval, 1972; unpublished), personality traits (Snyder and Shenkel, 1976), group memberships (Snyder and Fromkin, 1977), and consumer behaviors (Lynn and Snyder, 2002). For instance, a large body of research has demonstrated that people with a high NFU desire scarce, novel, customized or unpopular products and are more likely to be attracted by unusual marketing strategies as well as to shop in less popular outlets (for a review, see Lynn and Snyder, 2002).

Uniqueness seeking has been widely considered a defining feature of individualism^1^ (e.g., Markus and Kitayama, 1991; Kim and Markus, 1999; Lynn and Snyder, 2002; Oyserman et al., 2002). Substantial supportive evidence has been documented. For instance, while cross-cultural comparisons revealed that Americans tend to value unique objects and visual representations (relative to common ones) more than do East Asians (e.g., Kim and Sherman, 2007; Ishii et al., 2013), Koreans devalue and avoid individuals exhibiting non-normative characteristics more than Americans (Kinias et al., 2014); More directly, Japanese and Malaysian consumers reported a lower NFU compared to their American counterparts (Burns and Krampf, 1992; Takemura, 2014). Cross-temporal analysis also indicated that as individualism increases, American parents have been increasingly giving their children uncommon names during 1880 and 2007 (Twenge et al., 2010) and Japanese parents have become more and more likely to give their children unique names by pairing common Chinese characters with uncommon pronunciations during 2004 and 2013 (Ogihara et al., 2015). Within China, the individual-level analysis also indicated that a high NFU was associated with people’s high individualistic orientation but low collectivistic orientation (Tsai et al., 2013).

### The Change of Culture and Psychology in China

Historically, China is a collectivistic-oriented society where people are embedded in strong social bonds with their relatives or close in-group members (Triandis, 1995; Oyserman et al., 2002). In the past decades, however, due to rapid and massive socioeconomic development, Chinese culture and people’s psychology have undergone profound changes. A salient shift is the rise of individualism. The first line of research illuminating this trend is based on self-reported surveys (for an exception, see Santos et al., 2017). For example, while some studies have examined self-reported individualism directly and found a rising individualism in China (Ralston et al., 1999; Taras et al., 2012), other studies have examined the change of specific self-reported values and found a number of individualism-related values such as autonomy have become increasingly popular in China (Yang, 1996; Xu and Hamamura, 2014). The second set of studies have focused on the change of psychology mirrored in cultural products such as Ngram books. These studies showed that the relative usage of singular first-person pronouns (e.g., “I” and “my”; Hamamura and Xu, 2015) versus plural first-person pronouns (e.g., “we” and “our”) and the prevalence of words related to individualistic values (e.g., autonomy and freedom; Zeng and Greenfield, 2015) have been increasing over the past decades.

Regarding the change of collectivism, however, the picture is much more complex. On the one hand, many collectivistic or traditional values have been declining, including obedience, loyalty, modesty, hierarchy within the family and so on (Yang, 1996; Xu and Hamamura, 2014; Zeng and Greenfield, 2015); on the other hand, some traditional values have been continuing, including family love, friendships, patriotism and so on (Yang, 1996; Xu and Hamamura, 2014; Zeng and Greenfield, 2015). Certain traditional values even have become more prevalent such as obligation (Zeng and Greenfield, 2015), filial piety and ancestral worship (Yang, 1996). This revealed complexity may be partly due to the persistence and variation of subsistence style (e.g., Talhelm et al., 2014).

Although a large body of research has examined the change of culture and psychology in China, no research has explored how Chinese NFU has changed. Two recent cross-cultural studies provided some indirect evidence suggesting a rise of Chinese NFU. In one study, Chinese college students were found to report a higher NFU than American college students (Bian and Forsythe, 2012). In another study, Chinese Generation Y consumers also manifest a higher consumer NFU than their American counterparts (Simmers et al., 2014). In both studies, the researchers speculated that socio-cultural changes may have led to an increasing NFU among today’s Chinese. Nevertheless, a direct cross-temporal examination of this issue is still lacking. We fill this research gap in the current study.

### Overview of the Current Study

In examining the impacts of socio-economic change, researchers have relied on subjective self-reports or/and analysis of objective cultural products (Morling and Lamoreaux, 2008). We employed both approaches to examine whether the NFU has been increasingly popular among Chinese people. In Study 1, we examined the change of Chinese NFU by investigating the reported NFU across different generations. If younger generations report higher NFU, one possibility is that the NFU has been rising in China. In Study 2, we investigated the change of the Chinese NFU by examining Chinese baby names. Examining the characteristics of human names across different historical periods is a valid way to study cultural changes over time. While naming babies serves as a special kind of cultural practice reflecting core cultural values (Lieberson and Bell, 1992), the names themselves play an important role in cultural transmission across generations (Twenge et al., 2010; Varnum and Kitayama, 2010; Grossmann and Varnum, 2015). Moreover, one’s name, as a basis of self-construction, also influences one’s attitudes, beliefs, and even life choices (e.g., Hoorens and Nuttin, 1993; Pelham et al., 2002; Nelson and Simmons, 2007). Therefore, names are viewed not only as a product of culture but also as a reflection of human psychology. In Study 2, we specifically examined the usage of unusual given names. Unusual given names have been linked to uniqueness seeking at the cultural level (Twenge et al., 2010) and to the NFU at the individual level (Snyder and Fromkin, 1980, p. 129–143; Zweigenhaft, 1981). Thus, by studying changes in usage of unusual names, we can infer the change in the NFU, both as a cultural value and as an individual personality trait.

## Study 1: A Self-Reported Survey

### Method

#### Ethics Statement

The Ethics Committee of the Institute of Psychology, Chinese Academy of Sciences provided approval for the study. Additionally, we obtained online informed consent from all participants prior to commencing the test.

#### Participants

A total of 580 participants were recruited from an online pool for a major research lab located in Beijing, China. They were asked to complete a 10-min survey in exchange for 5 Chinese Yuan. One participant who failed to complete the survey was excluded, leaving a final sample of 579 participants (male = 251; female = 328), ranging in age from 13 to 62 years (*M* = 36.20, *SD* = 12.43).

#### Procedures and Materials

The participants completed three questionnaires, including surveys on emotional well-being, relationship closeness, and need for uniqueness, in a random order.^2^ After completing the questionnaires, participants provided demographic information, including their year of birth, gender and level of education. We used four items to measure the NFU on a 5-point scale (1 = *not at all/never*, 5 = *very much/very often*) (Lynn and Harris, 1997). The items include: (a) I prefer being different from other people; (b) Being distinctive is important to me; (c) I have a need for uniqueness; and (d) I intentionally do things to make myself different from those around me (α = 0.80). As the scale was originally created in English, we applied the commonly used procedure of back-translation to ensure equivalency of meaning when we translated it into Chinese (Brislin, 1980).

### Results and Discussion

**FIGURE 1:**
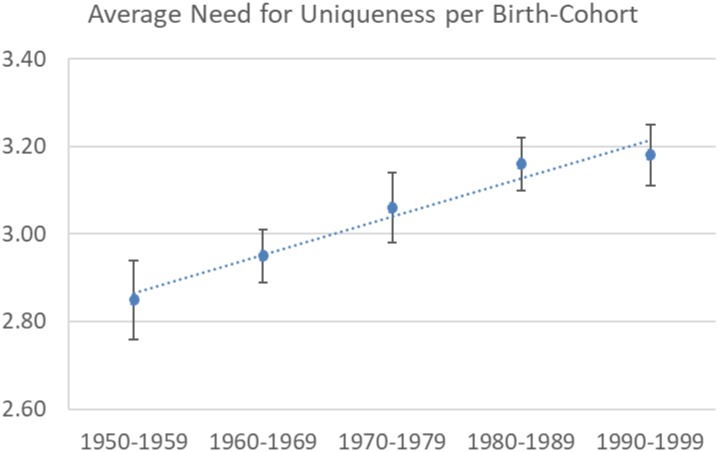
The reported need for uniqueness averaged for each of the five birth cohorts (Study 1).

Overall, the mean score of the NFU was 3.06 (*SD* = 0.76). Birth year was significantly correlated with both the NFU at the individual level (*r* = 0.15, *N* = 579, *p* < 0.001) and the average score of the NFU per year (*r* = 0.44, *N* = 50, *p* = 0.001). Both correlations suggested that younger generations reported a higher NFU than older generations (see **Figure 1**). Next, we regressed the birth year on the individual NFU rating while controlling for gender and educational level (1 = *elementary school*, 2 = *middle school*, 3 = *high school*, 4 = *bachelor*, 5 = *master*, 6 = *Ph.D.*). Again, our results confirmed a significant increase in NFU (*β* = 0.17, *t* = 3.94, *p* < 0.001). We detected no significant gender difference (*β* = 0.04, *t* = 0.87, *p* = 0.39). Education, however, mattered (*β* = .14, *t* = 3.21, *p* = 0.001), whereby participants with a higher education level reported a significantly higher NFU.

### Discussion

Study 1 showed that young people tend to have a higher NFU than older people, providing preliminary evidence for the increasing NFU in China. This finding, however, remains limited, as self-reports are subject to various methodology artifacts such as social desirability, response style, and reference group effect (Bachman and O’Malley, 1984; Paulhus, 1991; Heine et al., 2002). More importantly, cohort effects are confounded by age effects. In Study 2, we examined names of babies born in successive years over the past five decades. Testing for an increasing level of the NFU as reflected in unusual baby names allowed us to avoid the measurement problems associated with self-reports, while still controlling for age effects in examining cohort effects.

## Study 2: Analysis of Baby Names

### Method

Unlike the United States where the Social Security Administration’s database has documented baby names since 1880 or in other countries keeping public databases of names, no database of baby names is publicly available in China. Furthermore, to our knowledge, no prior research on baby names in China has ever been published. To build a sample of baby names in China, we collaborated with the Chinese Public Security Department which records all newborn baby names. Following our requirement, a computer scientist who was in charge of the name database in the Chinese Public Security Department performed the random sampling by using the syntax provided by the database management software. Eventually, we got 10 names per year from 1950 to 2009, resulting in a sample of total 600 names.

Because parents typically can only choose the given name for their babies and not the family name, we analyzed only given names. Notably, given names in China are not previously drawn from any general pool of names. Prior analyses of names in North American samples refer to extant popular name indexes (e.g., Jack or Emily as popular names; Twenge et al., 2010; Varnum and Kitayama, 2010; Grossmann and Varnum, 2015). In China, however, there are no such “established” names. Typically, a Chinese given name consists of either one or two characters based on the preference of the child’s parents or elderly family members. Thus, in our analysis, we test whether the given name consists of unique or rare (i.e., infrequently encountered) characters.

To identify the uniqueness of each character, we used the latest version of the *Modern Chinese Character Frequency of Use Dictionary* (*Xiandai Hanyu Zipin Tongjibiao*, 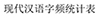, 1992) as a reference. The number listed in the dictionary for each character represents its frequency of usage per 1,000,000 characters. For a given name, we averaged the frequencies of each of its characters to represent the uniqueness of the name; we labeled this variable “name character frequency.” Two names revealed an extremely high frequency (more than 5 SDs away from the mean). Therefore, we excluded these two names from the analysis, making the final sample consist of 598 names (male: 327, female: 271).

### Results

**FIGURE 2:**
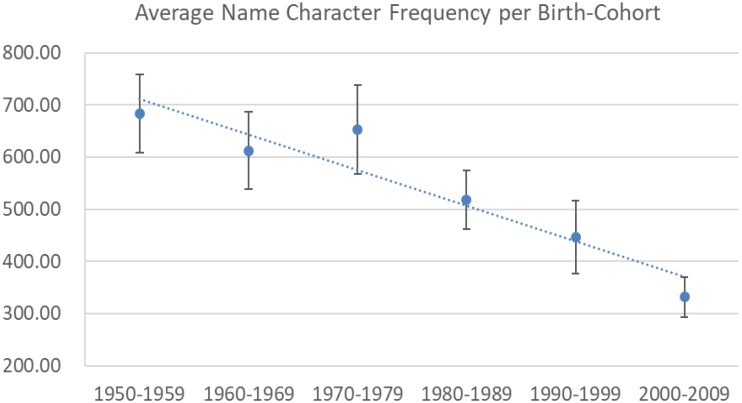
Average name character frequency averaged for each of the six birth cohorts (Study 2).

Overall, the average name character frequency across all individuals was 529.94 (*SD* = 692.57), while the average name character frequency per year across all years was 527.72 (*N* = 60, *SD* = 273.15). Birth year was significantly correlated with name character frequency at the individual level (*r* = -0.19, *N* = 598, *p* < 0.001) and the average name character frequency per year (*r* = -0.49, *N* = 60, *p* < 0.001). Both correlations supported a significant decrease in name character frequency since 1950 (see **Figure 2**). Next, we regressed the birth year on name character frequency while controlling for gender and name length (i.e., the total number of characters in the given name). Both gender and name length had a significant main effect. Specifically, girls’ names were more unique than boys’ names (*β* = -0.22, *t* = –5.72, *p* < 0.001), and on average, longer names were more likely to include high-frequency (“less unique”) characters (*β* = 0.19, *t* = 4.67, *p* < 0.001). Controlling for these two covariates, the effect of birth year remained significant (*β* = -0.15, *t* = -3.65, *p* < 0.001). In sum, a significant increase in need for uniqueness, as reflected in baby names, was evident.^3^

### Discussion

Study 2 extends Study 1 in several ways. First, the rising NFU reflected in names provides direct evidence for cohort difference. Second, the increasing prevalence of unique names suggests not only psychological changes but also cultural changes because names have been linked to both personality (Zweigenhaft, 1981) and cultural values (Twenge et al., 2010; Varnum and Kitayama, 2010). Somewhat unexpectedly, girls are more likely than boys to possess unusual given names. This result, however, is consistent with a prior study in which Varnum and Kitayama (2010) also found that girls were less likely than boys to receive popular names. They suspected that the gender effect might reflect parental expectations. In other words, parents might want their daughters to be more unique relative to their sons. Regardless, the change in name character frequency holds for both boys and girls in China.

## General Discussion

China has undergone unprecedented transformations in recent decades. In this research, we examined how NFU in China has changed. By examining self-reported NFU across different generations, Study 1 found that younger generations report a higher NFU than that of older generations. By studying the cultural product of baby names, Study 2 found that Chinese people have used increasingly unique names for their children over past 50 years, suggesting that the rising NFU among Chinese people is not due to age effects. Together, using both subjective and objective methodologies, our two studies yielded consistent results: the NFU, both as a cultural value and as a personality trait, is on the rise in China.

### Implications

Our research provides a close look at the changes in Chinese people’s psychology over the past decades through the lens of the need for uniqueness. Previous research has shown that social and economic changes have led to extensive cultural and psychological changes in China, among which important changes include the rise of many individualistic values as well as the decline of many traditional values (Hamamura and Xu, 2015; Zeng and Greenfield, 2015). In the current study, we reveal that another individualistic trait and value, NFU, has also been rising in China. This finding enriches our understanding of societal change’s psychological impact of societal change in China. As a personality trait, the revealed rise in the NFU is consistent with our prior finding that narcissism has been growing in China (Cai et al., 2011), given that the NFU has been linked to both narcissism (Emmons, 1984) and psychological entitlement (Campbell et al., 2004). As a cultural value, the recent rise in the NFU is in line with the previous finding that the cultural values of freedom and independence in China have been rising as well, while obedience has been on the decline (Xu and Hamamura, 2014; Zeng and Greenfield, 2015)—all being trends that are expected to cultivate the NFU (Lynn and Snyder, 2002). This trend in China further coincides with the upward trend of the NFU observed in the United States (Twenge et al., 2010) and Japan (Ogihara et al., 2015) where individualism has also been rising (Twenge et al., 2012; Grossmann and Varnum, 2015; Hamamura and Xu, 2015).

Conventional assumption holds that Chinese people, as an exemplar of collectivists, possess a lower NFU than Westerners. Recent studies, however, revealed that Chinese young people manifest an even higher consumer NFU than their American counterparts (Bian and Forsythe, 2012; Simmers et al., 2014). This finding is consistent with our result suggesting a growing trend of NFU in contemporary China. Our results also highlight the importance of considering culture as a dynamic system wherein established cultural differences across countries may vary with time, particularly for fast-changing societies such as China.

Given that the level of the NFU has been rising in China, people may infer further that the importance of the NFU has been rising. Although no direct examination of this issue has yet taken place, a number of studies do support this possibility, particularly in the domain of consumer behavior. For instance, in China, the NFU has been shown: (1) to predict online group buying (Zhang and Tsai, 2015), the pretentious consumption of luxury goods (Tsai et al., 2013), brand consciousness as well as the willingness to pay a price premium for name-brand merchandise (Chan and Wang, 2015); (2) to mediate the relationship between face consciousness and status consumption (Sun et al., 2015); and (3) to moderate the relationship between knowledge about luxury brands and attitudes toward the best-known brands (Zhan and He, 2012), as well as the relationship between hedonic (vs. utilitarian) goals and customized (vs. standardized) services (Ding and Keh, 2016). These findings are consistent with a large body of research on the relevance of the NFU to consumer behavior in the West (for a review, see Lynn and Snyder, 2002).

Our findings have various potential practical implications, particularly for managers in organizational and industrial areas as well as for officials in government ministries. First, since person-job fit is critical for employee job satisfaction and performance (Edwards, 1991), leaders and managers must be conscious of the rising trend of the NFU in China, particularly the high NFU among Chinese young people, and adjust the work environment to motivate people with an increasing NFU. In reality, they must alter many of their practices, including job design, employee hiring and training, and reward systems design, as all these might play a role in determining whether organizations can channel such a personal need for the collective good. Second, given that a high NFU is beneficial for creativity (Dollinger, 2003; Kim et al., 2013) and a challenging issue in contemporary China happens to be the improvement of creativity among employees and organizations, managers, government officials and policymakers may make use of the heightened NFU to promote Chinese creativity. Last but not least, the demonstrated importance of the NFU for consumer behavior suggests that there is a growing market for diverse and individualized products and services, which are beneficial for Chinese people to satisfy their increasing NFU. Relatedly, marketing campaigns that appeal to the NFU might become more effective, particularly in attracting young Chinese consumers.

### Limitations and Future Studies

Several limitations are notable. First, considering the large regional variation within China (e.g. Talhelm et al., 2014), the samples in our two studies, particularly baby name sample in Study 2, are relatively small, which may compromise the strength of our finding to some extent. Future replication may employ larger sample representing people from all areas in China; and if possible, also examine possible variations of the change within China. Second, we are limited in identifying the causes of the increasing tendency toward the NFU in China. Methodologically, it is difficult to identify the exact causes of cultural and psychological change because the changes usually occur naturally over the course of time, making it almost impossible to use an experimental or longitudinal design. Our current research designs also do not allow us to examine the antecedents of the rising NFU directly, but we can make some speculations. Since the NFU is an individualistic value and trait, the factors that may lead to the rise of individualism may also contribute to the NFU’s rise in China. Based on existing theories such as modernization theory (Inglehart and Baker, 2000) and social change and human development theory (Greenfield, 2009), many factors could have contributed to the rise of the NFU, such as increasing wealth, urbanization and education, decreasing family size, and others. Future studies may explore the unique contribution of each possible factor using a more sophisticated research design.

Some people may be interested in how important historical events, such as the Cultural Revolution of 1966–1976, may have influenced the NFU. Our study did not examine the details about the effects of such changes for two reasons. First, methodologically, our research design does not allow for us to pinpoint the influence of any specific social event because usually more than one factors may contribute to the change during a certain period^4^. Second, our goal is to delineate the overall trend of the NFU over the past several decades, rather than short-term changes caused by any particular event. In this regard, we think we have achieved our goal by revealing the rising trend of the NFU. Nevertheless, we acknowledge that important social events have the potential to produce a pronounced effect on cultural practice and even human psychology. For instance, one study reported that the Cultural Revolution influenced naming practices in Beijing and surrounding areas (Obukhova et al., 2014). Future studies may examine how social events could have impacted changes in Chinese culture and psychology during a specific historical period.

A large body of research in the West suggests that people may express and satisfy their NFU in a number of ways, such as through group identity and consumption (Lynn and Snyder, 2002). Burgeoning research in China has suggested that Chinese may express their uniqueness via creative or unpopular choices and special consumer behaviors (e.g., Tian et al., 2001; Tsai et al., 2013). Future studies may continue to examine other possible ways that are useful for Chinese to express their NFU, either in similar or dissimilar ways to those that Westerners practice.

## Author Contributions

HC proposed and designed this project. YF and YL collected the data. HC analyzed the data and wrote the initial draft. XZ and YJ provided critical feedback. HC and YJ revised the manuscript.

## Conflict of Interest Statement

The authors declare that the research was conducted in the absence of any commercial or financial relationships that could be construed as a potential conflict of interest.

## Footnotes

1 Here, we consider individualism as a type of cultural syndrome associated with many features (Triandis, 1995). For example, Triandis (1995) has identified 60 attributes that may be associated with an individualistic or collectivistic culture. Among them, need for uniqueness has been widely considered a central feature of individualist cultures (Markus and Kitayama, 1991; Kim and Markus, 1999; Oyserman et al., 2002).

2 The first two scales were designed for a different study. Thus, we exclude them in the analyses of this study.

3 The original distribution of name character frequency was highly skewed (skewness = 2.55, kurtosis = 8.21). We did a log transformation of the name character frequency, which led to a relatively normal distribution (skewness = -0.93, kurtosis = 1.23). We repeated all the analyses with the log-transformed frequency and found that all results remained similar.

4 We have tried to examine whether Culture Revolution period (1966–1976) manifested any unique contribution. We first created a dummy variable with “1” representing this period and “0” representing other years. We then regressed both birth year and this dummy variable onto NFU with gender and name length controlled. Results showed that while birth year was still significantly predictive (*β* = -0.19, *t* = -4.71, *p* < 0.001), the dummy variable was not (*β* = 0.04, *t* = 1.01, *p* = 0.31), suggesting that Culture Revolution period did not make unique contribution to the change of NFU.
